# Supplementary material for: Stacking Model‐Based Classifiers for Dealing With Multiple Sets of Noisy Labels
Source: Biom J. 2025 Mar 12;67(2):e70042. doi: 10.1002/bimj.70042 (PMC11898607; doi:10.1002/bimj.70042)
Supplement: Supplementary file 1 — Supporting Information [file BIMJ-67-e70042-s001.zip › density-based-ensemble-model-main/competitors_real_data/netlab-master/netlab-master/help/rbfgrad.htm]

Netlab Reference Manual rbfgrad


# rbfgrad

## Purpose

Evaluate gradient of error function for RBF network.

## Synopsis

```
g = rbfgrad(net, x, t)
[g, gdata, gprior] = rbfgrad(net, x, t)
```

## Description

`g = rbfgrad(net, x, t)` takes a network data structure `net`
together with a matrix `x` of input
vectors and a matrix `t` of target vectors, and evaluates the gradient
`g` of the error function with respect to the network weights (i.e.
including the hidden unit parameters). The error
function is sum of squares.
Each row of `x` corresponds to one
input vector and each row of `t` contains the corresponding target vector.
If the output function is `'neuroscale'` then the gradient is only
computed for the output layer weights and biases.

`[g, gdata, gprior] = rbfgrad(net, x, t)` also returns separately
the data and prior contributions to the gradient. In the case of
multiple groups in the prior, `gprior` is a matrix with a row
for each group and a column for each weight parameter.

## See Also

`rbf`, `rbffwd`, `rbferr`, `rbfpak`, `rbfunpak`, `rbfbkp`

---

**Pages:**
Index


---

Copyright (c) Ian T Nabney (1996-9)
